# Graph theoretic characterization of in vitro neuronal network development

**DOI:** 10.1186/1471-2202-15-S1-P207

**Published:** 2014-07-21

**Authors:** Uzair Khawaja, Tyler Stone, Lisa Morkowchuk, Thomas R Kiehl, Charles Bergeron

**Affiliations:** 1 Analytics Lab, Albany College of Pharmacy and Health Sciences, Albany, NY, 12208, USA; 2 Neural Stem Cell Institute, Rensselaer, NY, 12144, USA

## 

Multielectrode arrays (MEA) are routinely used to observe the development of neuronal networks in vitro, making simultaneous recordings of small proximal subpopulation activity within a cell culture. We propose the use of graph theoretic measures to deduce behavioral properties of neuronal networks as they develop in culture.

**Figure 1:**
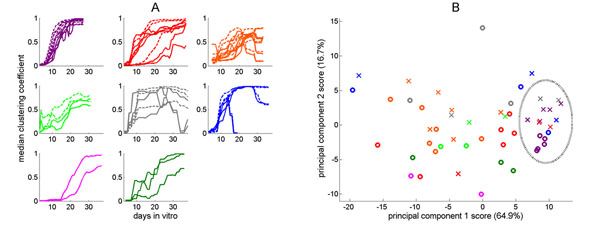
**(A)** Development signature of cultures, organized by batch. Solid curves indicate spontaneous mode and dashed curves indicate stimulated mode. Colors indicate batch number: **Batch 1 (purple)**, **Batch 2 (red)**, **Batch 3 (orange)**, **Batch 4 (light green), Batch 5 (grey)**, **Batch 6 (blue)**, **Batch 7 (pink)**, and **Batch 8 (dark green)**. Several cultures, including all cultures of batch 1, exhibit a *common sigmoidal development*. **(B)** PCA biplot showing 52 spontaneous (circles) and stimulated (crosses) cultures. The dotted ellipse denotes cultures exhibiting a *common sigmoidal development*.

We utilize 878 recordings from embryonic rat cortex cell cultures collected from 60-electrode, grid-type (200 μm) MEA’s [3]. We modeled each recording as a directed weighted graph with weights describing electrode connectivity. For each recording, we calculated each electrode’s clustering coefficient [1]. We averaged them over a sliding 5-day window, interpolating as needed and generating ~1700 features that describe each culture’s development (Figure 1A). Principal components analysis (PCA) [2] reduced the dimensionality of this feature space (Figure 1B). Batch 1 cultures possess a *common sigmoidal development*. Clustering coefficients rise sharply and a steady-state is observed with a fully clustered median electrode (Figure 1A). Stimulation appears to induce higher clustering more quickly and consistently. This similarity among batch 1 cultures is also evidenced as a tight grouping (Figure 1B). Several other cultures followed this development pattern; others presented different trends.

Each batch presents specific development signatures (Figure 1A) and a certain degree of consistency (Figure 1B). We believe that they capture meaningful biological variability. A greater number of cultures may yield richer and more robust characterizations. Our approach lends itself well to other downstream analysis methods, such as explicative classification models based on clustering coefficient profiles that characterize a tissue’s neuronal connectivity.
